# Prosocial Behavior in Toddlerhood: The Contribution of Emotion Knowledge, Theory of Mind, and Language Ability

**DOI:** 10.3389/fpsyg.2022.897812

**Published:** 2022-05-27

**Authors:** Elisa Brazzelli, Alessandro Pepe, Ilaria Grazzani

**Affiliations:** Lab for Developmental and Educational Studies in Psychology (https://www.labpse.it/en/), “R. Massa” Department of Human Sciences for Education, University of Milano-Bicocca, Milan, Italy

**Keywords:** prosocial behaviors, emotion knowledge, language ability, theory of mind, toddlerhood

## Abstract

While scholars have previously investigated the respective contributions of emotional knowledge and language ability to toddlers' prosociality, no studies to date have featured a battery of multiple direct measures assessing both of these abilities plus theory of mind on the one hand, and prosocial behavior on the other hand. In contrast, we conducted the present cross-sectional study with a view to evaluating the unique contributions of each of these three social cognition variables as antecedents of prosocial conduct during toddlerhood, measuring them via a series of individually administered standardized tasks. Furthermore, given that the existing literature documents mixed gender effects, we also set out to explore the role of gender in toddlers' prosociality. Finally, we also controlled for any effects of age on the patterns of association among the key variables. Participants were 127 children aged between 24 and 36 months (*M* = 29.2 months; *SD* = 3.5). We identified significant correlations among the variables under study. In addition, stepwise multiple regression analysis suggested that each of the social cognition (SC) abilities – i.e., emotion knowledge, theory of mind, and language - made a unique contribution to explaining variance in prosocial behaviors (PB). These findings show that SC is already associated with PB in toddlerhood and suggest the importance of fostering social cognition competence from the early years, with a view to increasing children's propensity to engage in prosocial conduct.

## Introduction

As part of a broader research program on the promotion of positive actions in toddlerhood, in the current study we investigated the contributions of emotion knowledge, theory of mind, and expressive and receptive language to prosociality in male and female toddlers. Although some studies have already investigated the contribution of social cognition and language to explaining the occurrence of prosocial behavior in toddlerhood, most of them were based on the observation of spontaneous behavior and/or indirect measures provided by caregivers; moreover, few have been used a multiple battery of direct measures to assess both socio-cognitive abilities (especially theory of mind) and the different types of prosocial conduct. The aim of our study therefore was to add empirical evidence on this topic that was based on individually administered, validated tasks.

Defined as voluntary actions intended to benefit another, prosocial behaviors have been linked to a variety of positive outcomes—at both the individual and interpersonal levels—in areas such as personal wellbeing, empathy, school achievement, social acceptance, and peer popularity (Eisenberg et al., 2015; Findley-Van Nostrand and Ojanen, 2018). The last two decades have seen an explosion of research on the genesis of early PB, including work examining its various forms (Dunfield, 2014), developmental trajectories (Dunfield and Kuhlmeier, 2013; Malti and Dys, 2018), socialization practices (Brownell, 2013; Brownell et al., 2013b), and both individual and gender differences (Hastings et al., 2007; Hine and Leman, 2013; Grazzani et al., 2017; Schachner et al., 2018). Nevertheless, few studies have investigated individual differences in the socio-cognitive abilities associated with frequency of prosocial behavior in toddlerhood, a period when all these skills undergo strong development. Furthermore, studies in this area have been mainly conducted using either indirect measures (e.g., questionnaires completed by adults) or observational studies in which the rate of occurrence of positive actions was not assessed.

Over the next paragraphs, we build up a rationale for the present research reviewing existing empirical findings on prosocial behaviors in the first years of life, the associations between prosocial behaviors and both emotion knowledge and theory of mind, and the role played by language ability in the development of children's prosociality. Finally, we present the mixed findings reported to date on the role of gender in the production of different prosocial actions. While focusing on the methodological aspects of the reviewed studies, we wish to provide a more accurate picture, as compared to the current literature, on the associations among the study variables.

### Prosocial Behaviors in Toddlerhood

One of the earliest types of prosocial behavior is instrumental helping, defined as helping another person to achieve an action goal. Helping emerges early in the first year of life, between 8 and 11-months-old, when infants become capable of independent locomotion (see Dahl, 2015; Köster et al., 2019). By the end of their first year, infants will assist experimenters by picking up items that are out of reach (Warneken and Tomasello, 2007) and pointing to sought-after objects that are out of sight (Liszkowski et al., 2008). By the middle of their second year, toddlers show instrumental helping by removing obstacles to help someone achieve a goal or by pointing out an alternative means of attaining an objective (Warneken and Tomasello, 2006). Such acts of helping are not only observed in laboratory settings: toddlers also help family members to accomplish simple goals in the home, for example by participating in chores (Zahn-Waxler et al., 1992; Dahl, 2015) and can share resources by giving up something of their own on behalf of another person in material need (Brownell et al., 2009, 2013a). At around 18–24 months of age, toddlers may be observed sharing food and toys, initially in response to the explicitly expressed desire of an interlocutor (Dunfield et al., 2011) and from 2 years of age spontaneously (Hay and Cook, 2007). Also, at the age of 2 years, children begin to display comforting behaviors in response to negative arousal on the part of another (Nichols et al., 2009), and responding in a variety of ways to the emotional needs they observe in those they interact with. Their reactions depend on, among other factors, their current level of social cognitive and prosocial maturity (Hoffman, 2000), as well as their degree of familiarity with interlocutors (Zahn-Waxler et al., 1992).

Children's repertoires of helping, sharing, and comforting behaviors diversify rapidly during late toddlerhood and the preschool years (Svetlova et al., 2010; Dunfield, 2014). This has led some researchers to explore individual differences in early prosociality, generating growing research interest in establishing the SC abilities that underlie the development and production of prosocial behaviors. In the next sections, we review existing findings on the contributions of emotion knowledge, theory of mind, and language abilities to children's prosocial behaviors, which are the factors we set out to investigate in this study.

### The Contribution of Emotion Knowledge to Prosocial Behavior

To carry out a prosocial action, a child must recognize that another person has a problem and understand how to resolve it (Vaish and Warneken, 2012; Brownell et al., 2013a; Dunfield, 2014; Grazzani et al., 2016b; Brazzelli et al., 2018). Therefore, the engagement in prosocial behaviors depends, at least in part, on an understanding of others' mental states, such as their emotions, desires, and intentions. Correlational studies have reported significant relationships between emotion knowledge (EK)—the ability to identify and understand emotions, including children's developing ability to recognize facial expressions, and label and understand causes of emotions (Saarni et al., 2008)—and specific aspects of prosociality. Ensor and Hughes (2005), in a small sample of 36 toddlers, examined the associations between toddlers' emotion understanding and positive behavior, as measured via an indirect measure, that is maternal ratings (Strengths and Difficulties Questionnaire; Goodman et al., 1997) and *via* video-based coding of the toddlers during 20-min' dyadic play with mothers and familiar peers. They found that maternal ratings of prosocial behaviors were positively correlated with performance on EK tasks, even after controlling for the effect of age. They also found that unique influence was not significant for verbal ability. We noted that 6 toddlers were aged 20–24 months, being too young to receive the EK task (Puppet task) selected by the researchers. In a longitudinal study from 2 to 4 years, Ensor et al. (2011) examined children's emotion knowledge as a predictor of prosocial action, finding that performance on emotion knowledge tasks at age 2 predicted observed helping and sharing behaviors at age 4, even after controlling for the effect of verbal ability. These findings were replicated by Eggum et al. (2011) in a study with preschoolers: the authors found that EK, measured at 42 months of age, was positively related to parent-reported prosocial orientation concurrently and over the following 1-year period. Denham et al. (2014) assessed 101 preschoolers via direct measures finding that emotion knowledge predicted participants' prosocial behavior. Similarly, Laurent et al. (2020) found that preschoolers' showing less emotion understanding show lower prosocial behavior as well, according to teachers' reports.

### Theory of Mind and Prosocial Behavior

Recognition of one's own and others' needs, desires, beliefs, and intentions is likely to facilitate prosocial behavior (Hay and Cook, 2007; Dunfield, 2014; Cigala et al., 2015). Hence, children with a better grasp of their own and others' internal states are expected to be more motivated to engage in prosocial actions both in the present and over time (Eggum et al., 2011), which in turn may lead them to develop a more sophisticated theory of mind (ToM). On investigating the relationship between ToM and prosocial behaviors, researchers have obtained mixed results, ranging from positive (Cassidy et al., 2003; Eggum et al., 2011; Wu and Su, 2014; Gross et al., 2015) to no correlations (Ruffman et al., 2006). However, we still know little about how ToM is linked with prosocial behavior in early childhood. Imuta et al. (2016) conducted a meta-analysis from 76 studies on children between 2 and 12 years of age, confirming that children who possess an advanced theory of mind are more likely to act prosocially. The relationship between ToM and prosocial behavior was independent of age and gender, although the association was stronger in children aged 6 and older and in girls. Importantly, this comprehensive meta-analysis revealed that only seven out of the 76 studies had been conducted with children younger than 3 years of age, and none had adopted standardized task of ToM.

### The Contribution of Language Ability to Prosocial Behavior

Language abilities play a prominent role in the development of emotional competence (Cassidy et al., 2003; de Rosnay et al., 2004; Ornaghi et al., 2019), as well as in the production of prosocial behaviors in early childhood (Barrett et al., 2007). In this field of research, children's language ability has been studied via measures of either receptive (the ability to comprehend language) or productive language (the ability to produce language). Longitudinal studies have found that early language abilities positively influence the development of prosocial conduct. For example, Girard et al. (2017) set out to identify the directional associations between expressive language ability and prosocial behavior between 3 and 5 years of age in a sample of 14,004 children and their families, enrolled on the UK Millennium Cohort Study. Children's expressive language and prosocial behavior were assessed via standardized tools and parent reports, respectively. The results showed that more advanced expressive language at 3 years of age was associated with increased prosocial behaviors by 5 years. In another longitudinal study with 547 typically developing children in Germany, Rose et al. (2018) investigated the effects of 3-year-old children's language comprehension and production on parent- and teacher-rated cooperative behavior, physical aggression, and emotional self-regulation over a 4-year period. Path models showed that receptive but not productive language significantly predicted the development of cooperative behavior when key child and family characteristics (e.g., SES, non-verbal cognitive abilities, and early cooperation at age 3 years) were controlled for. Finally, Conte et al. (2018) found, based on naturalistic observations, that, in toddlerhood, more advanced language abilities were significantly associated with a greater tendency to share resources with peers: the authors argued that receptive language abilities may both facilitate children's understanding of others' mental states and improve their ability to share efficiently with others.

### Gender Differences in Prosocial Behaviors

With regard to the role of gender in early prosocial behavior (PB), empirical studies have typically indicated that girls both perform more prosocial behaviors and are judged more prosocial than boys (Eisenberg and Fabes, 1998; Hine and Leman, 2013; Bouchard et al., 2020), and also express more empathy and prosocial orientation (Zahn-Waxler et al., 1992; Tisak et al., 2007; Rhee et al., 2013; Girard et al., 2017; Conte et al., 2018). In their cross-sectional study, Ensor and Hughes (2005), with their small sample, found no significant gender differences for sixteen of eighteen prosocial measures; one exception (girls “often volunteers to help others”) was marginal and obtained via indirect measures. In qualitative terms, female PB is described as more compassionate and sympathetic, while male prosocial behavior can be perceived as more agentic, engaged, and active (Hastings et al., 2007). Furthermore, with respect to the influence of research methods, studies that draw on self- and other-report instruments yield considerably larger effect sizes for gender differences in PB than do those that use observational methods (Eisenberg and Fabes, 1998). These variations suggest that the stereotypical belief that girls are more prosocial than boys may influence reports of prosocial behavior. This belief may, in turn, act as a socializing force that creates and sustains both actual gender differences in behavior and the stereotype itself. The implication is that gender differences in PB may emerge because of gender-differentiated socialization practices: in other words, parents and other agents of socialization may put more pressure on girls to be responsive to the physical and emotional needs of others (Hay and Cook, 2007; Eisenberg et al., 2015).

## The Present Study

Overall, the existing literature suggests that emotion knowledge, theory of mind, and language ability are all positively associated with prosocial behavior from preschool age onwards. Nevertheless, few studies have evaluated the associations of these socio-cognitive abilities with prosocial conduct in toddlerhood as well, especially with regard to ToM. In the present study, we wish to explore whether the propensity to act prosocially is also associated with emotion knowledge, theory of mind, and language in even younger children. Indeed, previous studies have mainly investigated prosocial manifestations via parent-report measures or observations of spontaneous prosocial behaviors. The former approach bears the risk of social desirability effects that may bias responses; the latter is undermined by the low and random frequency with which children engage in prosocial action in uncontrolled natural settings. In this study, by using a standardized battery of tasks to assess both SC skills and PB, we wished to exert systematic control over the conditions under which children's propensity to act prosocially is assessed.

Hence, the main goal of this cross-sectional study was to add further evidence to the existing literature on the respective associations between EK, ToM, and language abilities and toddlers' prosocial behaviors by assessing children's performance on a battery of socio-cognitive and prosocial tasks. All toddlers were presented with the same tasks so as that we could associate for each child the presence or absence of prosocial actions with their social cognition scores. In keeping with the existing literature, we expected to find positive statistically significant associations among the variables under study. In particular, we expected that EK, ToM, and language abilities would be associated with a higher rate of prosocial conduct. Furthermore, given that previous studies on the role of gender yielded mixed findings, we set out to examine the contribution of this variable to toddlers' prosociality. Finally, we also controlled the pattern of association among variables for the effect of age.

## Method

### Participants

Participants were 127 toddlers (62 girls, 48.8%) whose mean age was 29.2 months (*SD* = 3.5; range: 24–36 months). The children were Italian native speakers whose linguistic development fell within the normative standards for their age group as emerged assessing their language skills. They were attending ten different nurseries (early childhood education centers) in the North of Italy, which were all under the same management and shared the same educational programs. All children were from low or medium socioeconomic status (SES) backgrounds. Most of their parents held a high school diploma (85% of mothers and 70% of fathers) and were in white-collar employment (51.4% of mothers and 41.5% of fathers). Other parents were manual workers (20.8% of mothers and 42.5% of fathers), executives or self-employed professionals (13.4% of mothers and 19 of fathers), while the remainder were unemployed (14.4% of mothers and 5.8% of fathers). In terms of family composition, only children accounted for 34.4% of the sample, 44.8% had one sibling, 16% had two siblings, and the remaining 4.8% had three or more siblings. Prior to commencement of the study, all the children's families were provided with a detailed written brief about the research and asked to provide written consent for their children to participate. The research design was approved by the Ethics Committee of the University of Milano-Bicocca (protocol n. 348/2018). The study was conducted in accordance with the ethical principles and code of conduct of the American Psychological Association.

### Measures

The children individually completed a battery of instruments in a quiet room at their own nurseries. Overall, the assessment lasted ~20 min (range: 16–27 min), and the tasks were presented in counterbalanced order. Two different female experimenters administered the tasks in which they had received in-depth training. Each session was videotaped and subsequently coded by trained researchers who were blind to the children's performance on the other study measures. Intercoder reliability for each task was established by an independent coder who scored at least 25% of the recordings from the full sample.

#### Prosocial Behavior

**Figure 1:**
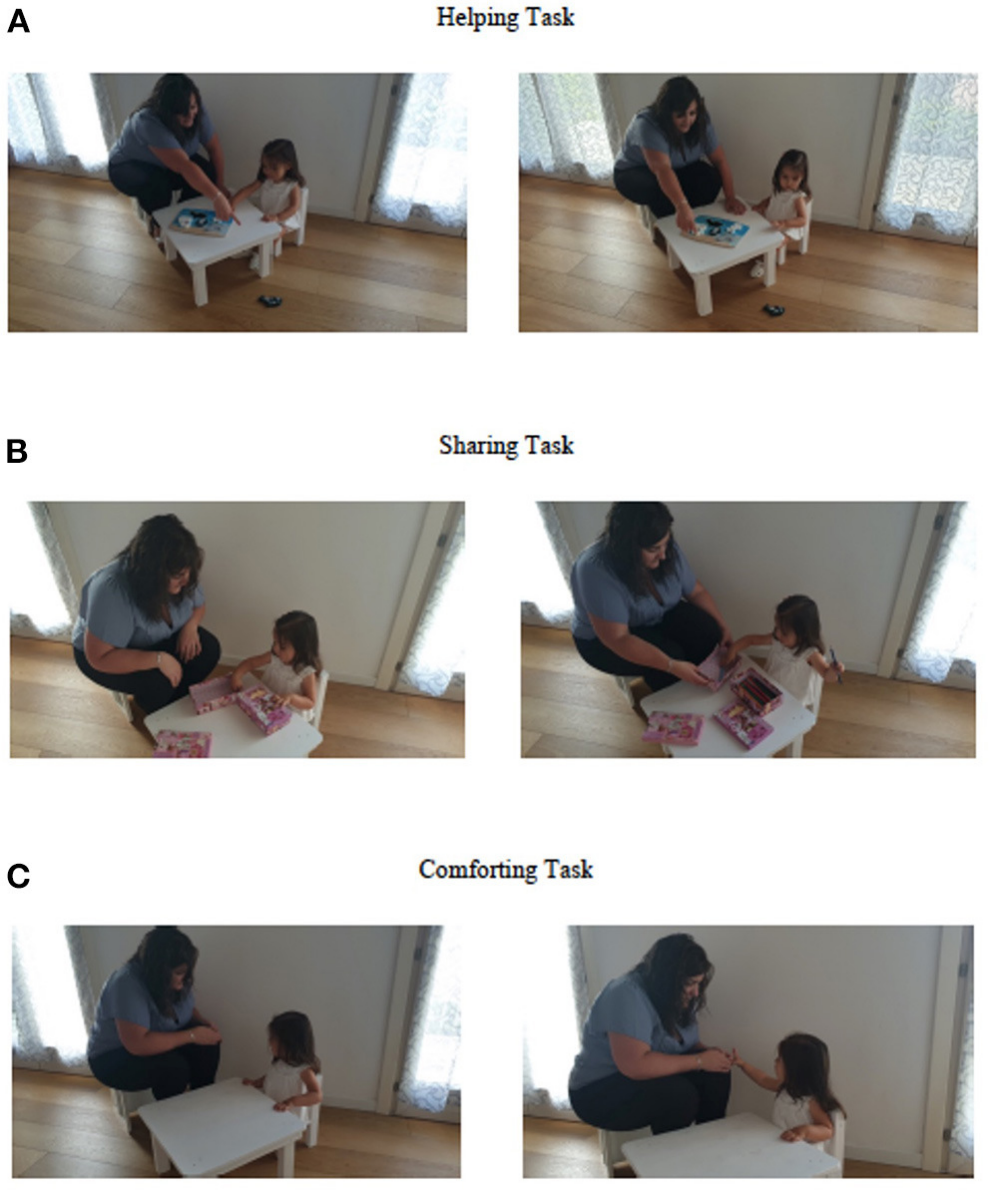
Illustrations of the Italian prosocial task battery (PBT-I). **(A)** Helping task. **(B)** Sharing task. **(C)** Comforting task.

We administered the Italian Prosocial Task Battery (PBT-I) (adapted from Warneken and Tomasello, 2006, 2007; Dunfield and Kuhlmeier, 2013) to assess the toddlers' propensity to engage in a variety of prosocial behaviors. Different experimental situations were devised using techniques previously developed by the authors and applied in Brazzelli et al. (2021). The battery, described in detail in the article by Brazzelli and Grazzani (in press), consisted of two helping tasks, two sharing tasks, and two comforting tasks. Participants were brought in one at a time and seated opposite a female experimenter at a small table positioned in the middle of a dedicated testing room at the nursery (see Figure 1), where they were presented with the battery.

For the helping tasks, we deployed close replicas of Warneken and Tomasello's (2006) “out-of-reach” task and Dunfield and Kuhlmeier's (2013) “across-the-room” task. In the “out-of-reach” task, the experimenter picked up a small plastic toy (a card in the post-test session), playfully “walked” it across the table, but then dropped it over the far edge while vocalizing “Oops!”. The experimenter then reached toward the toy with an outstretched arm and hand. For the first 5 s after the toy fell, the experimenter focused her gaze on the toy. Then for five more seconds, she alternated her gaze between the toy and the child, until the child provided a response, or the trial ended. Trials ended when a total of 10 s had elapsed. The experimenter never directly asked the child for help. The “across-the-room” task was similar to the out-of-reach task, with the key distinction being that the jigsaw puzzle piece that the experimenter wished to recover was already at the other side of the room, and thus the child had never observed it in the experimenter's possession. When the child and experimenter had completed most of the puzzle, the experimenter would exclaim, “We're missing a piece!” The experimenter would then look around the room and, spotting the piece of the puzzle, say “Oh!” and reach toward the piece. Again, at this point, the experimenter looked directly at the puzzle piece for the first 5 s and then for the subsequent 5 s alternated her gaze between the puzzle piece and the child.

In the sharing tasks, participants were presented with two distinct scenarios: the first involved sharing food, whereas the second involved sharing toys. In both tasks, the experimenter received an empty container while the participant received a box full of food or toys (colored pencils). Before giving the child its container, the experimenter showed her own container to the toddler and said, “Look what I have”. The child was then given his or her container. The experimenter then made a sad face and held out her hand with the palm facing upwards. For the first 5 s, the experimenter focused on her own container, and for the subsequent 5 s, alternated her gaze between her box and the participant. The experimenter never verbally requested food or pencils. The experimenter acknowledged receipt of the items with a neutral “Thank you”.

In the comforting tasks, participants were presented with two varieties of distress: physical and emotional distress. In the first, the experimenter banged her knee off the edge of the table, which in turn hit a metal bracket, making a loud noise. The experimenter then sat down with a look of distress on her face. She rubbed her knee, vocalizing pain (e.g., “Oh! My knee, I banged my knee”). For the first 5 s, the experimenter focused on her knee, before alternating her gaze between her knee and the participant for another 5 s. The experimenter never directly requested aid. The emotional comforting task was administered similarly to the physical trial. The experimenter showed the child her favorite toy. While the experimenter was playing with her toy, she tore a hole in the back of it. The experimenter looked at her toy and exclaimed in a sad voice “Oh! My toy, I broke my toy!” For the first 5 s thereafter, the experimenter looked at her toy with a sad expression on her face, and for the subsequent 5 s she alternated her gaze between the toy and the child.

Each prosocial task was coded for the target behavior following the coding scheme used in Ketelaar et al. (2015). Specifically, 2 points were awarded for displaying the correct target behavior during the first 5 s, 1 point was awarded for producing the correct target behavior during the next 5 s, and 0 points were awarded for displaying any non-target behavior. Each child was assigned a total score ranging from 0 to 12. To assess inter-rater reliability, 30% of the prosocial tasks were coded by both judges; inter-rater agreement between them was j =.90. The reliability coefficient for the overall measure was α = 0.79.

#### Emotion Knowledge

To assess the children's understanding of basic emotions, we used the validated Italian version of the Affect Knowledge Test (AKT; Denham, 1986; Camodeca and Coppola, 2010). The materials are two puppets with blank faces and four felt discs, each depicting a facial expression corresponding to a distinct basic emotion (happy, sad, angry, afraid/scared). Given the young age of the participants, we only administered three subtasks from the battery: the expressive task, the receptive task, and the situation task. The expressive task considers the ability to label emotions, asking children to verbally name, one by one, the emotions depicted on four faces. The tester points each face and asks the child to label them, using the prompt question: “How does he/she feel?”. Next, in the receptive task the experimenter shuffles the faces and asks the child: “Where is the [emotion] face?”. The child is invited to non-verbally identify, by pointing, the four emotional faces, coherently with verbal labels provided by the tester. After this session, the child is trained, that means the tester labels and shows the facial expression of each emotion, exaggerating gestures, vocal expression, and body language. The aim is to teach emotions, in preparation for the next parts of the test. The situation task requires the child to identify the emotional face shown by the main puppet, male or female according to the child's gender. It consists of eight scenarios, in which the puppet feels happy, sad, angry, or scared. The social situations acted out with puppets by the examiner in these vignettes are typical, which means the puppet feels an emotion that commonly most children experience in such situation. For instance, John experiences anger at having a block tower destroyed by Paul, that is a typical emotion felt by children who are subjected to this provocation. Participants received a score of 2 for a correct response, 1 for an incorrect response that is within the same emotional valence (e.g., “good” or “smiling” for Happy, “crying” for Sad, “scared” for Angry, etc.), and 0 for a completely inappropriate response (e.g., “Happy” for Angry, “surprised” for Scared, “sad” for Happy, etc.). Each child received a total score ranging from 0 to 32, and three sub-scores relative to the three sections administered (expressive task: max. 8; receptive task: max. 8; situation task: max. 16). The internal consistency coefficient for the three scales taken together was α = 0.90. Internal consistency coefficients for the individual sub-sections of the instrument were: α = 0.69 for the expressive task, α = 0.71 for the receptive task, and α = 0.70 for the situation task.

#### Theory of Mind

To assess children's theory of mind, we used a battery comprising the Desire-Action and Desire-Emotion tasks (Wellman and Woolley, 1990). Identifying suitable ToM tasks to administer to toddlers is challenging for researchers. This is because toddlers are in between the age at which ToM is investigated by means of implicit tasks and preschool age, when children may be appropriately administered the typical verbal false belief task battery. The tasks we adopted offer a valuable means of measuring ToM in toddlers specifically and allow us to access and evaluate children's ability to predict a story character's actions and emotional reactions, as well as their understanding of the role of desires in mediating emotional reactions.

Children made judgments about the actions and emotional reactions of small cardboard characters in each of three types of situation: the Finds-Wanted situation (the character wants something that may be in either one of two locations, searches at Location 1, and retrieves desired object), the Finds-Nothing situation (identical to Finds-Wanted except that the character finds nothing upon searching Location 1), and the Finds-Substitute situation (identical to Finds-Wanted, except that upon searching Location 1, the character finds an object different to the desired one). The children are invited to make action judgments by predicting the character's subsequent action, that is to say, whether he or she will go on to search in Location 2 or alternatively stop searching. An understanding of the implications of characters' desires should lead to a prediction of continued searching in the Finds-Nothing and Finds-Substitute scenarios but not in the Finds-Wanted situation. The participants are then invited to make emotion judgments by characterizing the character's emotional reaction in terms of whether he or she will be happy or sad. An understanding of the role of desires in mediating emotional reactions should yield a prediction of happiness in the Finds-Wanted situation but sadness in the Finds-Nothing and in the Finds-Substitute situations. Participants received a score of 1 for a correct response and 0 for a wrong response, yielding a total score of up to 6. Inter-item reliability coefficients for the overall scores based on the two tasks was 0.299.

#### Language

The Picture Naming Game (PiNG; Bello et al., 2012) was administered to provide a measure of children's verbal ability. The PiNG is a robust, standardized, Italian-language tool for the direct observation of language skills in children between 19 and 37 months of age. It provides a measure of young children's semantic competence by evaluating both their receptive and their expressive lexical knowledge. PiNG consists of four subtests: Noun Comprehension (NC), Noun Production (NP), Predicate Comprehension (PC) and Predicate Production (PP), each of which comprises 20 lexical targets with two training items. Both comprehension subtests (NC and PC) include a target word, a semantic distracter (corresponding to the target for the production subtests) and another distracter that is not semantically related to the target. We applied the standard scoring procedures outlined in the PiNG Manual. The reliability of the language measures was assessed by calculating Cronbach's alpha. For the overall measure, the reliability was 0.890. Reliability coefficients for the sub-scales (i.e., nouns and predicates) were 0.820 and 0.765, respectively.

### Data Analysis

The minimum number of participants required was determined by an a priori power analysis (using the Gpower software package; Faul and Erfelder, 1992) with statistical significance set at 5%, power at 95%, and a medium-sized effect (around 0.30) expected. The outcome of the analysis was that a sample of at least *N* = 111, with 109 degrees of freedom, was required for this study.

At a first stage of analysis, descriptive statistics (means, standard deviations, and indexes of score distribution), and zero-order correlations were computed for the children' prosocial behavior, language ability, theory of mind, and emotion knowledge scores. Mean scores for the prosocial behavior were the following: 1.61 for helping (SD = 1.60), 0.88 for sharing (SD = 1.30) and 0.30 for comforting (SD = 0.93). At the second stage of our data analysis, we evaluated the unique contributions of the different variables (especially in terms of explained variance and standardized beta weights) as antecedents of prosocial behaviors in children. To this end, we computed a stepwise multiple regression (Maxwell, 2000), with the overall score for prosocial conduct as the target variable of the regression. Before performing the regression analyses, we checked our data set for appropriateness and compliance with statistical assumptions (see Ornaghi et al., 2016). Our model was set as a three-step multiple regression, with age and gender entered at the first step, emotion knowledge (EK), theory of mind (ToM), and language ability (LANG) at the second step, and the effect of the interactions between the different antecedents (EK^*^ToM, EK^*^LANG, and ToM^*^LANG) at the third and final step. We compared the different steps of the regression model, testing for significant variation in explained variance in the specified dependent variable at each step and examining the statistical significance of the standardized beta weights. Finally, we computed the variance inflation factor (VIF) to test whether the data met the assumption of collinearity, taking a VIF value above a cutoff point of 4 to indicate that multicollinearity was a concern (Dias and Castro, 2011). All variables used in the regression analyses were centered to enhance the interpretability of the data and reduce potential issues linked to multicollinearity.

The data were also checked for rates of missing values and the presence of normality violations (Fiorilli et al., 2017) in conflict with the assumptions of multiple regression (i.e., multivariate normality). We identified no missing values or violations of normality in terms of excessive kurtosis or skewness. Finally, we adopted the Mahalanobis distance criterion (*p* < 0.001) to check for outliers. Four multivariate outliers were identified (3.14%) and omitted from the analysis. As a result, the regression analysis was performed on 123 valid cases. We also applied a bootstrap non-parametric resampling procedure. Bootstrapping procedures can compensate for the limitations of statistical methods that assume standard distribution (for details, see Shrout and Bolger, 2002) in small to moderate sample sizes (*N* < 500, Hoyle and Kenny, 1999). Thus, a bootstrap analysis with 2,000 bootstrap sample simulations was conducted to obtain estimates of the beta weights with their 95% confidence intervals.

## Results

### Descriptive Statistics and Zero-Order Correlations

To provide a first comprehensive overview of prosocial scores in association with aspects of SC, Table 1 summarizes the main descriptive statistics for emotion knowledge, theory of mind, language skills, and PB, as well as the zero-order correlations among these variables.

**Table 1:** Main descriptive statistics and zero-order correlations.

	**1**	**2**	**3**	**4**	**5**	**6**
1. Age	–					
2. Gender	0.026	–				
3. Prosocial Behaviors (PB)	0.322**	0.040	–			
4. Theory of Mind (ToM)	0.468**	0.126	0.532**	–		
5. Emotion Knowledge (EK)	0.457**	0.084	0.538**	0.615**	–	
6. Language Ability (LANG)	0.555**	0.014	0.536**	0.630**	0.657**	–
Mean Scores (M)	29.3	–	2.82	1.58	10.1	208.1
Standard Deviation (SD)	3.5	–	2.83	1.52	6.44	93.9

*Note: Gender is a dummy variable (neither M nor SD values can be reported)*.

In general terms, the zero-order correlational analysis revealed strong, statistically significant patterns of association between emotion knowledge, theory of mind, and language abilities. Similarly, prosocial behaviors were positively correlated with language abilities (*r* = 0.536), emotion knowledge (*r* = 0.538) and theory of mind (*r* = 0.532) scores. Interestingly, the zero-order correlations also revealed that age was more strongly associated with language ability (*r* = 0.555) than with theory of mind (*r* = 0.468) and emotion knowledge (*r* = 0.457) scores. Finally, participants' gender was not correlated with any measure. The results of the correlational analysis provided support for conducting a multiple-regression analysis.

### Results of the Stepwise Multiple Regression on Prosocial Behaviors

The target variable in the multiple stepwise regression model was the cumulate score for prosocial behaviors. Children's age and gender were entered together at Step 1. At this stage, the model was statistically significant, *F*_(2, 120)_ = 7.02 *p* < 0.001, and accounted for approximately 9% of explained variance. However, while age made a significant contribution to explaining variability in prosocial scores [β = 0.321, *p* < 0.001; 95% CI (0.131–0.738)], gender did not [β = 0.032, *p* = 0.715; 95% CI (−0.729 to 1.170)]. At Step 2, *F*_(5, 117)_ = 13.89, *p* < 0.001, entry of the scores for AKT (measure of emotion knowledge), ToM, and LANG led to a statistically significant increase (Δ*F* = 16.63, *p* < 0.001) in explained variance (ΔR2 = 0.268) with overall explained variance for the model increasing to R2 = 0.346. Specifically, EK [β = 0.244, *p* = 0.024; 95% CI (0.016–0.203)] was found to display a statistically significant and positive association with prosocial behaviors, as was both ToM [β = 0.241, *p* = 0.020; 95% CI (0.074–0.802)] and LANG [β = 0.235, *p* = 0.043; 95% CI (0.002–0.013)]. All three antecedent variables displayed a similarly sized association with prosocial outcomes. Interestingly, at Step 2, the association between prosocial behaviors and children's age was not statistically significant, leaving the other variables as the main contributors to prosocial scores. Finally, at Step 3, when the interactive effect was entered in the regression model, *F*_(8, 114)_ = 8.71, *p* < 0.005, the equation remained statistically significant but the interaction's unique contributions to the regression model were not statistically significant (i.e., there was no statistically significant increase in explained variance, ΔR2 = −0.007). Further details of the regression model results are summarized in Table 2.

**Table 2:** Results of regression analysis and coefficients of ToM, EK and LANG on prosocial behaviors.

	**Step1**				**Step2**				**Step3**			
	**β**	***p***	**LOW**	**UP**	**β**	***p***	**LOW**	**UP**	**β**	***p***	**LOW**	**UP**
Age	0.321	0.001	0.131	0.378	−0.032	0.723	−0.158	0.109	−0.043	0.637	−0.175	0.102
Gender	0.032	0.715	−0.729	11.170	−0.007	0.929	−00.860	0.798	−0.017	0.821	−0.935	0.799
EK					0.244	0.024	0.016	0.203	0.311	0.143	−0.016	0.285
ToM					0.241	0.020	0.074	0.802	−0.096	0.801	−1.516	1.051
LANG					0.235	0.043	0.002	0.013	0.249	0.099	−0.001	0.014
EK*ToM									−0.168	0.472	−0.001	0.001
EK*LANG									0.306	0.557	−0.004	0.008
ToM*LANG									0.114	0.715	−0.054	0.062
R2	0.090	0.346	0.336									
F for R2	7.02	16.64	0.423									
variation	(*p* = 0.001)	(*p* < 0.001)	(*p* = 0.737)									

*Note. β: standardized beta weight; p: statistical significance, LOW: lower bound for confidence interval; UP: upper bound for confidence interval*.

## Discussion

In the current cross-sectional study, we set out to add new evidence to the literature on the associations between socio-cognitive (SC) variables and the occurrence of prosocial behavior (PB) in early childhood. Existing studies on this topic have in fact mostly been conducted with children of preschool age. Furthermore, with regard to toddlers, the scant findings reported have been predominantly obtained via indirect measures or under non-controlled conditions, or even with a small sample (Ensor and Hughes, 2005). In contrast, in this study, 127 toddlers individually completed a battery of direct measures, thus allowing us to assess their emotion knowledge, theory of mind, and language ability on one hand and their prosocial behaviors on the other. In fact, since all toddlers were presented with the same opportunities to act prosocially or otherwise, we could associate, for each participant, the presence or absence of prosocial behavior with their social cognition scores.

Correlational and stepwise multiple regression analyses of the data yielded two main findings, which we shall discuss in turn. First, we found statistically significant associations both among the socio-cognitive variables under study on one side, and between PB and EK (*r* = 0.538), PB and ToM (*r* = 0.532), and PB and language ability (*r* = 0.536), respectively, on the other side. Second, regression analysis showed that EK, ToM, and language skills together explained a substantial amount of variance (about 35%) in the occurrence of prosocial actions.

With regard to the first outcome, associations among SC variables as well as between SC and PB have been scarcely investigated in toddlers. In the present study, the strong associations among the variables under study (see Table 1) confirm that aspects of social cognition skills (namely EK, ToM, Language) are also strongly interrelated in children between 2 and 3 years of age. This is the age when emotional and cognitive perspective-taking grow considerably (Dunfield, 2014), young children become more aware of others' inner states (Schachner et al., 2018) and the comprehension and production of language undergo rapid development (Girard et al., 2017). As far as we know, no studies have investigated these SC variables in toddlerhood, as the few available findings include EK and Language but do not include ToM (Ensor and Hughes, 2005; Ensor et al., 2011). The correlational findings of this study are in keeping with the outcomes of studies with toddlers (e.g., Ensor et al., 2011; Grazzani et al., 2018) documenting correlations between verbal ability and emotion understanding, and with preschoolers (Eggum et al., 2011). However, the correlations outcomes reported by Ensor et al. were rather modest, suggesting the hypothesis that their findings reflect the contrast between direct but partially controlled observation on one side, and indirect information from maternal ratings on the other side. In addition, in their longitudinal study, Eggum et al. found that EK and ToM were positive related within and across time, but unlike what was done in the present study they did not take into account the verbal ability variable.

Whereas, the SC variables here investigated were positively associated with age, none of them were associated to gender variable. Significant, important differences in social cognition among young children as a function of gender are not documented and this suggests that at least during the first year of life the development of SC is generally the same in girls and boys (Hughes, 2011) even if parental and teachers' socialization practices may start to influence children's development (Song et al., 2022). In studies from preschool onwards, when gender has been controlled for, it has been found to make little or no contribution to explaining variance for aspects of SC, for instance of EK. In a fairly recent study with a sample of 3- to 8-year-old children, Fidalgo et al. (2017) found that emotion understanding was unaffected by gender in relation to eight out of the nine components assessed.

With regard to the target variable, that is to say, the manifestation of PB in the experimental setting, the correlational findings showed significant associations between PB on one hand and EK, ToM, and language, respectively, on the other hand. These associations were stronger than the relationship between prosocial behavior and age (*r* = 0.32), although this relationship was significant too. These findings are in line with those by Ensor et al. (2011) who showed robust associations between EK and PB as well as between verbal ability and PB. In their cross-sectional study, Ensor and Hughes, 2005 found that PB (rated by mothers) was associated to EK even when age effects were controlled.

With respect to the second outcome, the regression analysis allowed us to identify the unique contributions of the individual social cognition abilities to explaining variance in prosocial behavior. In this regard, we obtained a robust outcome: emotion knowledge, theory of mind, and language together explained some 35% of the variance in toddlers' manifestation of prosocial actions, with a medium effect size that corroborates the interest of these findings. As far as we know, this is one of the very few studies that has shown the role of EK, ToM and language as indicators of prosocial behavior as early as toddlerhood. Indeed, to behave prosocially (e.g., helping someone or sharing a toy with someone) a child must recognize that another person has a problem and need something, putting oneself in the other's perspective (Brownell et al., 2013a), that is using her skills of SC. Therefore, the engagement in prosocial behaviors at this age depends on an understanding of others' mental states. Studies by Ensor and colleagues, who turned their interest to SC associated to PB, showed the role of EK and Language in explaining PB but did not include theory of mind variable in their research design.

The regression analysis (see step 2) confirmed the lesser role not only of gender but also of age in explaining variance in PB. The age variable reduced its significant role as an indicator of the incidence of prosocial behavior when the socio-cognitive variables under study were taken into consideration. Similarly, no interactive effect among the variables under study (see Step 3) emerged to provide a different interpretation of the variance in PB, given that neither EK^*^ToM nor EK^*^language nor ToM^*^language were significant. Overall, these findings, based on explained variance and standardized beta weights, support the hypothesis of the unique role of Language as antecedents of PB as well as of the other SC variables. These findings are in line with those by Cassidy et al. (2003). In contrast, in their cross-sectional study Ensor and Hughes (2005) found that unique predictive effects were significant for EK but not verbal ability. A possible explanation lies in the small size of the sample (36 toddlers) which did not allow to carry our statistical analysis (e.g., regression analyses) capable to produce completely reliable results.

In addition, regression analyses showed that gender was neither an antecedent nor a predictor of children's propensity to act prosocially. We consider this an interesting result because it is based on the administration of direct measure, and not on indirect measures (e.g., parents' and teachers' ratings). It confirms the outcomes from Ensor and Hughes (2005) and from Drummond et al. (2017) who, studying the socio-moral emotions of guilt and shame in 30-month-old children, did not find any difference of PB as a function of gender.

Overall, our findings with toddlers may offer a more comprehensive picture of the relation between the main aspects of SC and the presence of PB, as well as of the not significant role of gender with regard to the study variables. This findings add information to the current scant literature, for instance providing new data on the association between toddlers' theory of mind and prosocial conducts.

### Conclusions, Limitations, and Educational Implications

This study, bringing together outcomes on early SC and early social competence, examined the respective contributions of emotion knowledge, theory of mind, and language ability to explaining prosocial behaviors in toddlerhood, thereby adding key evidence to the literature on the socio-cognitive factors associated with prosociality in 2- and 3-year-olds. In general, we found that not only EK and Language are indicators of PB in toddlerhood, as investigated in previous studies, but so is theory of mind variable; and that gender was not significantly associated with the study variables.

Nevertheless, this research is not without its limitations. First, its cross-sectional design precluded the identification of cause-effect relationships among the variables, as well as limiting the extent to which the findings may be generalized. Second, although the size of the sample was adequate, it was not large enough to allow statistical analysis as a function of the different types of prosocial actions (e.g., helping vs. comforting), which we view to be a promising future line of research (e.g., Newton et al., 2016). Third, the study did not examine the role of additional factors (Song et al., 2022), such as warmth, prosocial socialization practices, or attachment security, in explaining individual differences in prosocial conduct.

Despite these limitations, the present findings offer evidence in support of promoting prosociality from early childhood. Specifically, they suggest the importance of fostering children's socio-cognitive competences from the first years of life with a view to also boosting their early development of prosocial skills. Recent findings (see for example (Grazzani et al., 2016a; Malti et al., 2016; Spinrad and Gal, 2018; Brazzelli et al., 2021) show that the promotion of social cognitive skills at nursery (for example, by reading stories enriched with mental state language and conversing about inner states) can enhance perspective-taking and language abilities, as well as prosocial behaviors in young children, and should therefore be pursued by policymakers and practitioners working in educational settings.

## Data Availability Statement

The raw data supporting the conclusions of this article will be made available by the authors, without undue reservation.

## Ethics Statement

The studies involving human participants were reviewed and approved by University of Milano-Bicocca. Written informed consent to participate in this study was provided by the participants' legal guardian/next of kin.

## Author Contributions

EB contributed to designing the study, collecting the data, and writing the manuscript. AP made a key contribution to analyzing and interpreting the data. IG contributed to designing the study, interpreting, discussing the data, and revising the manuscript. All authors contributed to the article and approved the submitted version.

## Funding

This study was funded by a doctoral grant awarded to EB by the University of Milano-Bicocca and by a grant from the University of Milano-Bicocca (2019-ATE-0193) awarded to IG.

## Conflict of Interest

The authors declare that the research was conducted in the absence of any commercial or financial relationships that could be construed as a potential conflict of interest.

## Publisher's Note

All claims expressed in this article are solely those of the authors and do not necessarily represent those of their affiliated organizations, or those of the publisher, the editors and the reviewers. Any product that may be evaluated in this article, or claim that may be made by its manufacturer, is not guaranteed or endorsed by the publisher.
